# Graph structure based data augmentation method

**DOI:** 10.1007/s13534-024-00446-4

**Published:** 2024-11-19

**Authors:** Kyung Geun Kim, Byeong Tak Lee

**Affiliations:** 1 VUNO Inc., 479, Gangnam-daero, Seoul, 06541 Korea; 2 Medical AI Co., Ltd., 163, Yangjaecheon-ro, Seoul, 06302 Korea

**Keywords:** Data augmentation, Graph structure, Medical waveform data, Robustness

## Abstract

In this paper, we propose a novel graph-based data augmentation method that can generally be applied to medical waveform data with graph structures. In the process of recording medical waveform data, such as electrocardiogram (ECG), angular perturbations between the measurement leads exist due to imperfections in lead positions. The data samples with large angular perturbations often cause inaccuracy in algorithmic prediction tasks. We design a graph-based data augmentation technique that exploits the inherent graph structures within the medical waveform data to improve the F1 score by 1.44% over various tasks, models, and datasets. In addition, we show that Graph Augmentation improves model robustness by testing against adversarial attacks. Since Graph Augmentation is methodologically orthogonal to existing data augmentation techniques, they can be used in conjunction to further improve the final performance, resulting in a 2.47% gain of the F1 score. We believe that our Graph Augmentation method opens up new possibilities to explore in data augmentation.

## Introduction

Data augmentation techniques are easy and intuitive methods to promote generalization, widely used in many fields of deep learning, especially in computer vision [1, 2]. Applying augmentations to image data is intuitive since it is not difficult to recognize the effect of an augmentation operation on the label change. This is mainly because humans are naturally domain experts in image recognition tasks. However, when dealing with medical waveform data, it is very difficult or even impossible to perceive the altering effect of an augmentation scheme on the label across the entire dataset. While only limited understandings of medical waveform data are available at the human level, the lead-based measurement procedure introduces inherent graph structures in that data [3]. To elaborate, a lead-based measurement procedure measures the electrical activity of an organ from multiple positions in space. Because the electrical potential at close locations is similar, in fact, a function in positional difference, these relations fundamentally embed graph structure within the measurement. From these observations, we propose a novel, nontrivial data augmentation scheme for medical waveform data that exploits these underlying graph structures.

This paper’s main ideas and experiments were developed using electrocardiogram (ECG) datasets as a concrete example. Still, it is not very difficult to see the generalizability of the suggested Graph Augmentation scheme to other medical waveform datasets involving similar measurement structures. In recording 12-lead ECG signals, an ideal location exists for each lead with fixed angular positions between them [4]. These leads, as a whole, collectively measure the three-dimensional electrical activities of the heart. Due to this fact, the similarity between the measurements taken from the leads is higher if the leads are physically closer. Therefore, a graph structure naturally arises by the relative positions between the leads. We take advantage of these graph relations to propose an augmentation technique that introduces perturbations to a measurement taken from one lead using signals measured on the other leads.

Many existing augmentation methods in signal processing are no different from those in computer vision, in addition to other methods tailored for this domain. Specifically, the augmentations in the form of transformations in the time or frequency domain and inserting random noises are typically applied [5, 6]. These methods are also popular in ECG tasks as many works in the literature apply some modification or an extension of these methods in signal processing [7–9]. While these augmentation methods are helpful, they cannot provide perturbations that exploit the existing graph structure of the data. Since our method could generate augmentations that introduce perturbations with respect to the graph relations of lead measurements, we propose that our method could reduce the overfitting of the trained networks to graph-structure-oriented noises, resulting in a better performance. In addition, we show that the trained networks also become more robust to perturbations generated by adversarial attacks to demonstrate achieved robustness. Another advantage of our method is that because the augmentation is done methodologically orthogonal to the traditional augmentation methods, they could have an additive effect on the performance. We demonstrate the effectiveness of the proposed approach on diverse prediction tasks using three different datasets.

The main contributions of our proposed methods include: (1) We introduce a new, generalizable Graph Augmentation technique that can be applied to any models across various tasks involving medical waveform datasets. (2) The proposed data augmentation method can enhance model robustness and overall performance. (3) We opened up a new possible direction in data augmentation technique to explore.

## Methods

### Graph augmentation design

In order to construct an augmentation graph, we first examine how 12-lead ECG signals are measured and processed. The 12-lead ECG is defined by aVR, aVL, aVF, I, II, III, and V1-V6 leads, measured by 6 precordial and 4 limb electrodes. The leads can be interpreted as real or virtual positions in space to measure three-dimensional electrical activities generated by the heart [10]. Naturally, since the electrical potentials measured at two close locations must be similar, the signal similarity very much depends on the physical relative locations of each lead. We used this similarity information to represent the graph structure between the 12 ECG leads. Specifically, the average correlation (cross-correlation at lag $k=0$) of the lead measurements is computed to construct the weighted adjacency matrix $\textbf{A}$ as computed below:

1 $$\begin{aligned} \textbf{A}_{ij} = \mathbb {E} \left[ {\frac{\sum _{t=1}^{T} {(X^{(i)}_t - \mu ^{(i)}) (X^{(j)}_t - \mu ^{(j)})}}{\sigma ^{(i)} \sigma ^{(j)}}}\right] , \ \ i \ne j \end{aligned}$$

where $\textbf{X}^{(i)}, \textbf{X}^{(j)}$ are the signal vectors of length *T* measured at leads *i* and *j*. For the case $i = j,\ \textbf{A}_{ij} = 0$ is chosen.

Using the graph defined by $\textbf{A}$ as the augmentation graph, a linear combination of the other leads could be constructed for one specific lead *i*:

2 $$\begin{aligned} \mathbf {\widetilde{X}}^{(i)} = \sum _{j \ne i} {\textbf{A}_{ij} \textbf{X}^{(j)}} \end{aligned}$$

**Fig. 1:**
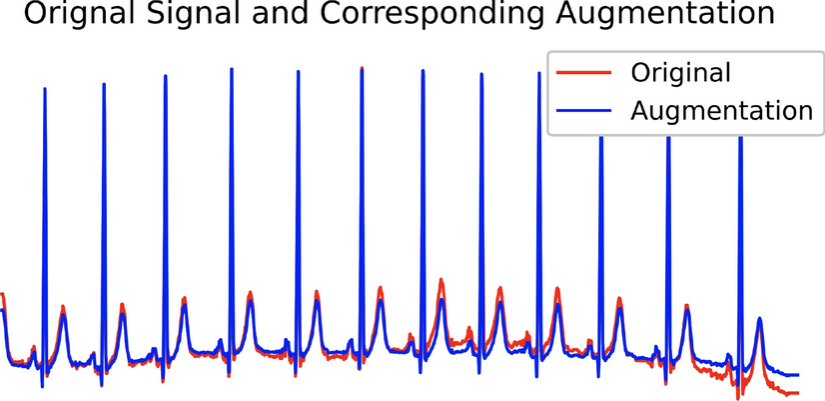
The comparison between an original signal and its corresponding augmentation

The constructed signal $\mathbf {\widetilde{X}}^{(i)}$ combines the signals measured from other leads according to how similar they are to lead *i*. Therefore, it is reasonable to think of $\mathbf {\widetilde{X}}^{(i)}$ as a graph structure-induced augmentation. To illustrate the similarity, example of such augmented signal $\mathbf {\widetilde{X}}^{(i)}$ and original signal $\textbf{X}^{(i)}$ is shown in Fig. 1.

Using the augmented signal $\mathbf {\widetilde{X}}^{(i)}$, a convex combination between the original signal and the final augmentation at some lead *i* is computed:

3 $$\begin{aligned} \mathbf {\widehat{X}}^{(i)} = (1-\lambda ) \textbf{X}^{(i)} + \lambda \mathbf {\widetilde{X}}^{(i)}, \ \ \lambda \in [0, 1] \end{aligned}$$

The augmentation is randomly applied with probability of *p* where parameter $\lambda$ is drawn from a uniform distribution $\mathcal {U}(0, \alpha )$ every time $\mathbf {\widehat{X}}^{(i)}$ is computed. The augmentation parameters *p* and $\alpha$ are hyperparameters to be tuned.

### Augmentation combination details

In order to compare our augmentation method to other methods, we selected several popular existing augmentation techniques. The parameter combinations determining how each augmentation is applied are tuned using RandAugment [11] for peak performance. In order to apply RandAugment [11], an intensity parameter shared across all of the augmentation schemes must be defined. Let $\gamma$ be an intensity parameter shared across every augmentation scheme. With $\gamma$, we applied the described augmentations according to the following rules. (1) Adding a Gaussian noise for every time point drawn i.i.d from $\mathcal {N}(0, \gamma )$ [12]. (2) Transformation in the time domain where $\gamma$% of the signal is randomly cut or zero-padded to be expanded or compressed using bilinear interpolation to fit the original length [13]. (3) Smoothing using weighted moving average window defined by Gaussian kernel with length, $l \in \{1, 2, 3, 4, 5\}$ which is chosen with equal probability according to $\gamma$ [14]. (4) Masking operation in which $\gamma$% of the signal is masked with zero starting at a position chosen uniformly random [15].

**Fig. 2:**
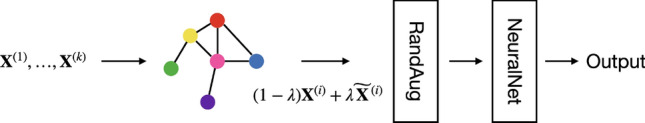
The overall structure of augmentation module

When applying Graph Augmentation and traditional augmentations simultaneously, we first apply the Graph Augmentation followed by the traditional augmentations. The order in which augmentations are applied first is critical for the following reasons. Traditional, lead-wise perturbations are done using different strategies, meaning that if the Graph Augmentation is applied afterward, a single trace of a waveform will contain a mix of multiple augmentations at the same time, possibly becoming too noisy. More importantly, the correlation values of the augmentation graph will no longer be valid after normal augmentations are applied. Therefore, applying traditional augmentations before the Graph Augmentation will have an adverse effect on the performance of the neural networks to be trained. The overall structure of the augmentation module is presented in Fig. 2.

## Experiments

### Datasets and models description

The experiments are carried out using three different open databases of The China Physiological Signal Challenge 2018 (CPSC) [16], SHAOXING [17] and Physikalisch Technische Bundesanstalt-XL (PTBXL) [18] which includes 7,166, 10,615 and 21,837 data points each. Each dataset includes labels for different types of arrhythmia. CPSC and PTBXL datasets contain labels fit for mult-label tasks for the following arrhythmia detection: atrial fibrillation, first-degree atrioventricular block, left bundle branch block, right bundle branch block, premature atrial contraction, premature ventricular contraction, ST-segment depression, and ST-segment elevated for CPSC and normal ECG, myocardial infarction, ST/T change, conduction disturbance and additionally, hypertrophy for PTBXL. SHAOXING dataset contains labels fit for multi-class classification tasks of seven classes composed of sinus bradycardia, sinus rhythm, atrial fibrillation, sinus tachycardia, atrial flutter, sinus irregularity, and supraventricular tachycardia. The training, validation, and testing sets are divided within the same datasets into 70%, 15%, and 15%, respectively.

To investigate the efficacy of the proposed method, we tested on the well-known models: Residual Network (ResNet) [19], Efficient Network (EfficientNet)  [20] and Densely Connected Network (DenseNet) [21]. Since these networks were originally designed for low-resolution image classification, we modified them for usage in 1D signals. For ResNet, we implemented a modification of ResNet that is popularly used for handling ECG data [22]. To make correct adjustments for the length of the signal, the pooling layer of the last block is modified from 2 to 1. For EfficientNet, we implemented a modified version of EfficientNet-B0. Specifically, 2-dimensional $N \times N$ filters are changed to 1-dimensional $N^2$ filters, and stride operation is added at the 5th stage. For DenseNet, we set the growth rate to 12 and the filter size to 16 for the entire network, where each block consists of 6 bottleneck layers. The networks are trained using Adam optimizer with a learning rate of 0.001. We used dropout regularization with $p=0.1$ and a batch size of 32. $l_2$ regularization with a coefficient of $10^{-5}$ is applied.

### Performance evaluation and comparison

In order to evaluate the performance of our augmentation method, we first compared the performance gain of Graph Augmentation against no augmentation as well as normal augmentations. We also show that the Graph Augmentation method can be applied on top of normal augmentations by showing that they have an additive effect on performance. The results of these experiments are summarized in Table 1 where each entries are F1 scores averaged over multiple repeated experiments. Although performance gain achieved from normal augmentations comes from optimal parameter combination tuned by RandAugment, Graph Augmentation, and normal augmentations show similar performance gain on average across every model and dataset. Additionally, when Graph Augmentation and normal augmentations are applied at the same time, additional performance gain has been observed across every dataset and model. As a result of these experiments, we arrived at the following conclusions. First, we show that using Graph Augmentation alone has a similar impact on network performance than using not only one but multiple well-tuned normal augmentation methods. Second, we conclude that performance gain from Graph Augmentation is orthogonal to normal augmentations, resulting in an additive effect on performance.

**Table 1:** Performance of neural network models for each dataset trained according to different augmentation schemes

	Residual network				Efficient network				Densely connected network			
	Base	RandAug	GraphAug	Both	Base	RandAug	GraphAug	Both	Base	RandAug	GraphAug	Both
CPSC	0.7896	0.8020	0.8032	**0**.**8043**	0.7570	0.7864	0.7750	**0**.**7953**	0.7869	0.7924	0.7932	**0**.**8014**
PTBXL	0.7654	0.7675	0.7688	**0**.**7718**	0.7450	0.7543	0.7488	**0**.**7566**	0.7580	0.7623	0.7645	**0**.**7659**
SHAOXIN	0.8718	0.8805	0.8854	**0**.**8909**	0.8605	0.8976	0.8814	**0**.**9041**	0.8556	0.8698	0.8727	**0**.**8769**

Bold denotes the highest values in each experiment

### Augmentation parameters optimization

Depending on how the augmentation parameters are tuned, they can have critical effects on the performance of the trained networks. For maximal performance gain, we have tuned these parameters for both normal and Graph Augmentations using RandAugment. We observed that, for the normal augmentations, the following RandAugment scheme clearly outperforms hand-searched parameter combinations.

**Fig. 3:**
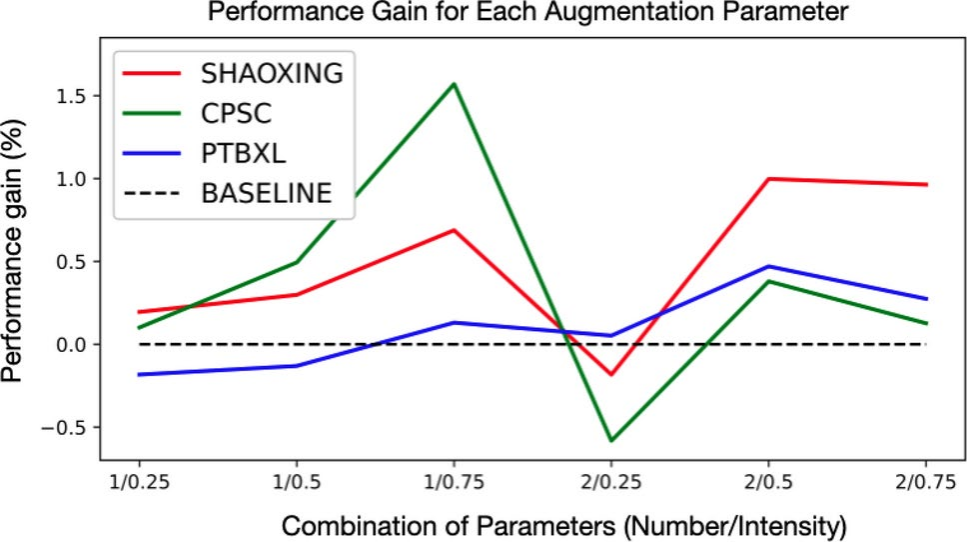
The performance gain in terms of the F1 score of ResNet trained with six different augmentation parameter combinations for RandAugment. The number and intensity parameters represent the input parameters to RandAugment, which are explored with grid-search

We have tried six different augmentation parameter combinations, measuring the performance gain achieved for each combination. As the summary of performance gain for ResNet shows in Fig. 3, performance gain achieved by augmentation methods is usually quite marginal. In addition, applying multiple augmentations on top of each other does not guarantee performance gains [11]. However, since the performance gain resulting from Graph Augmentation is on par with RandAugment, we conclude that it can provide a considerable amount of performance gain compared to other single augmentation methods.

### Robustness effect of graph augmentation

We hypothesized that the main reason for the performance gain achieved by the Graph Augmentation method lies within the network’s robustness with respect to graph structure-induced perturbations. To elaborate, if the network trained with Graph Augmentation is robust to these perturbations, it will make more correct predictions on the perturbed data samples when tested. To show the network robustness against graph perturbations, testing datasets with large graph structure-based perturbations is required. An ideal way to build such a test set is to have lead-wise, positional perturbations at the measurement level. Since this is intractable, we instead constructed perturbed data samples using adversarial attack method [23] which finds an (sub) optimal perturbation to change the label of the data as best as possible. Therefore, by demonstrating adversarial robustness, we believe sufficient evidence is provided to explain the performance gain achieved by Graph Augmentation. In addition, we believe that the Graph Augmentation and normal augmentation schemes have an additive effect on performance for the following reasons. In contrast to Graph Augmentation, the input data generated by normal augmentations introduces perturbations limited to each *waveform* rather than the relations between. As a result, the normal augmentation reduces overfitting related to noises within each waveform signal, which is independent of the effect of the Graph Augmentation. For this reason, the two different augmentation methods implement robustness that is orthogonal to each other, having additive effects when applied at the same time.

**Fig. 4:**
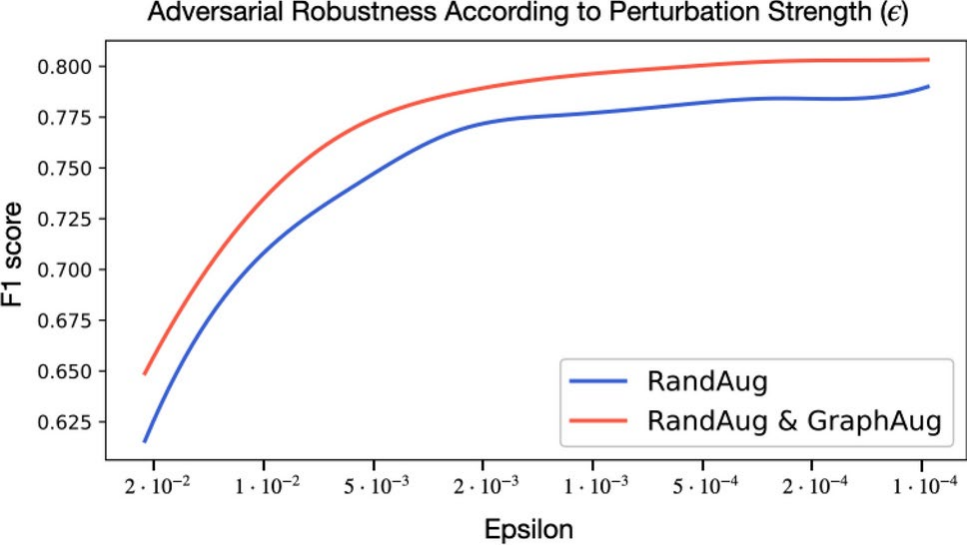
The adversarial robustness of ResNets trained using RandAugment and using both RangAugment and Graph Augmentation according to different perturbation strength ($\epsilon)$

In the robustness experiments, we used ResNet trained on the CPSC dataset evaluating the F1 scores according to the perturbation strength $\epsilon$. We evaluated the effect of the Graph Augmentation by comparing the F1 score decay of the network trained with only RandAugment and the network trained with Graph Augmentation added on top. As shown in Fig. 4, the network trained with both augmentations is more robust by a significant margin for every $\epsilon$ tested. Thus, we conclude that the Graph Augmentation can offer robustness with respect to the adversely constructed perturbations.

## Discussion

### Effectiveness and performance of graph augmentation

To demonstrate the effectiveness and validity of Graph Augmentation, we conducted extensive performance tests across all combinations of three widely used datasets and three distinct neural network architectures. Each experiment was averaged over multiple trials to minimize performance variance due to stochasticity in the training process. The prediction tasks, as detailed in Sect. 3.1, were selected to be as broad as possible, ensuring that the results are not biased toward specific tasks.

For performance evaluation, we employed a range of classic data augmentation techniques from time series and signal processing, as described in Sect. 3.2. Instead of applying all classic augmentations simultaneously, we used RandAugment to optimize augmentation combinations, tailoring them for maximum performance in each model and dataset pairing. As presented in Table 1, Graph Augmentation, despite being a single augmentation method, performs comparably to the RandAugment-optimized multi-augmentation techniques. This result is notable, as it shows that one augmentation method can achieve similar competency across various datasets and models as a tailored combination of augmentations for each pairing. Additionally, our results emphasize that further performance gains can be consistently achieved by combining classic augmentation methods with Graph Augmentation.

### Implication of graph augmentation

Our work’s primary contribution is its unique perspective. Rather than focusing solely on the time series aspect of the ECG signals, we shifted our attention to how they are measured. For instance, the ECG is measured through the 12-lead system, which essentially captures 12 points in space that measure the heart’s electrical activity during beating cycles. Graph Augmentation leverages the imperfections in the precision of these locations from patient to patient. The core idea behind Graph Augmentation is to amplify these imperfections through carefully designed perturbations rather than attempting to eliminate them. Applying augmentation derived from these locational imperfections makes the model trained with Graph Augmentation more robust to them. While there is no straightforward way to prove this experimentally, we believe the performance gain achieved with the robustness experiment can provide valuable insights into the underlying phenomena.

Another significant advantage of Graph Augmentation over waveform-based augmentations is its structurally lower probability of label modification. For instance, if we were to add a sinusoidal wave to an ECG waveform as an augmentation, the label could be changed from normal ECG to atrial flutter, depending on the type and magnitude of the added wave. While this kind of label shift can be detected when each ECG signal is examined individually, it is almost impossible to detect systematically across multiple ECG signals. On the contrary, Graph Augmentation is structurally robust to label change because it is mainly the linear combination of the same electrical activity measured from different leads. Therefore, it is easier to systematically control the augmentation intensity to minimize label shifts while preserving the effect of the augmentation.

### Limitations and future work

While the Graph Augmentation method has proposed a novel way to interpret 12-lead ECG recordings as graph structure data, our work has a few limitations. In this work, we deliberately proposed the simplest and the most intuitive way to construct the graph structure that embeds 12-lead-based ECG recording criteria. We believe a more sophisticated method can better represent the relations between the ECG recordings from different leads than taking the average correlation. One future research direction might be constructing a simple model with a decent representation of the dynamics between the signals recorded from each lead. Additionally, the experiments provided in this research only involve 12-lead ECG data out of many medical time series data that measure the electrical activity of an organ in multiple directions in space. Extending from our foundational intuition of Graph Augmentation, we believe that with some theoretical enhancement, Graph Augmentation can be generalized to other forms of medical time series data. Despite these limitations, our work with Graph Augmentation presents an effective augmentation method that opens up a novel direction for exploring graph-structured medical time series data. This potential for future exploration and discovery should inspire optimism about graph-structured-based augmentation methods.

## Conclusion

In this work, we propose a novel data augmentation method called Graph Augmentation that exploits underlying graph structures of medical waveform data. By using average correlations between the signals recorded from each lead, approximations of recordings from 12-lead ECG signals are constructed. We used these with the original signal, and taking a convex combination, we successfully introduced augmentations that have minimal effects on the labels of each data sample. We show the effect of the Graph Augmentation method in three different open datasets and three different neural network models. In addition, we also show the effect of Graph Augmentation on the network’s robustness with respect to adversarial attacks. A critical extension we would like to show in our future work is how our method generalizes to other data formats, such as electroencephalograms. As the direction of our future work suggests, we have introduced a new direction to explore in data augmentation methods for datasets that have underlying graph structures.

## References

[1] Shorten C, Khoshgoftaar T. A survey on image data augmentation for deep learning. J Big Data. 2019;6:1–48. 10.1186/s40537-021-00492-0 PMC8287113 34306963

[2] Iwana BK, Uchida S. An empirical survey of data augmentation for time series classification with neural networks. 2020; arXiv preprint arXiv:2007.15951 10.1371/journal.pone.0254841 PMC8282049 34264999

[3] Jekova I, Krasteva V, Leber R, Schmid R, Twerenbold R, Müller C, Reichlin T, Abächerli R. Inter-lead correlation analysis for automated detection of cable reversals in 12/16-lead ECG. Computer Methods Progr Biomed. 2016;134:31–41. 10.1016/j.cmpb.2016.06.003. 10.1016/j.cmpb.2016.06.003 27480730

[4] Wilson FN, Johnston FD, Rosenbaum FF, Erlanger H, Kossmann CE, Hecht H, Cotrim N, de Oliveira RM, Scarsi R, Barker PS. The precordial electrocardiogram. Am Heart J. 1944;27(1):19–85. 10.1016/S0002-8703(44)90603-4.

[5] Wang F, Zhong S-H, Peng J, Jiang J, Liu Y. Data augmentation for EEG-based emotion recognition with deep convolutional neural networks. In: Schoeffmann K, Chalidabhongse TH, Ngo CW, Aramvith S, O’Connor NE, Ho Y-S, Gabbouj M, Elgammal A, editors. MultiMedia Model. Cham: Springer; 2018. p. 82–93.

[6] Ko T, Peddinti V, Povey D, Seltzer ML, Khudanpur S. A study on data augmentation of reverberant speech for robust speech recognition. In: 2017 IEEE international conference on acoustics, speech and signal processing (ICASSP), 2017; pp. 5220–5224. IEEE

[7] Nonaka N, Seita J. Data augmentation for electrocardiogram classification with deep neural network. 2020; https://arxiv.org/abs/2009.04398

[8] Raghu A, Shanmugam D, Pomerantsev E, Guttag J, Stultz CM. Data augmentation for electrocardiograms 2022; https://arxiv.org/abs/2204.04360

[9] Pan Q, Li X, Fang L. In: Liu, C., Li, J. (eds.) Data augmentation for deep learning-based ECG analysis, 2020; pp. 91–111. Springer, Singapore. 10.1007/978-981-15-3824-7_6 .

[10] Bear LR, Cheng LK, LeGrice IJ, Sands GB, Lever NA, Paterson DJ, Smaill BH. Forward problem of electrocardiography: is it solved? Circ Arrhythm Electrophysiol. 2015;8(3):677–84. 25834182 10.1161/CIRCEP.114.001573

[11] Cubuk ED, Zoph B, Shlens J, Le QV. Randaugment: practical automated data augmentation with a reduced search space. In: Proceedings of the IEEE/CVF conference on computer vision and pattern recognition workshops, 2020; pp. 702–703

[12] Um TT, Pfister FM, Pichler D, Endo S, Lang M, Hirche S, Fietzek U, Kulić D. Data augmentation of wearable sensor data for Parkinson’s disease monitoring using convolutional neural networks. In: Proceedings of the 19th ACM international conference on multimodal interaction, 2017; pp. 216–220

[13] Le Guennec A, Malinowski S, Tavenard R. Data augmentation for time series classification using convolutional neural networks. In: ECML/PKDD workshop on advanced analytics and learning on temporal data 2016 Sep 19

[14] Nguyen T-S, Stüker S, Niehues J, Waibel A. Improving sequence-to-sequence speech recognition training with on-the-fly data augmentation. In: ICASSP 2020-2020 IEEE international conference on acoustics, speech and signal processing (ICASSP), 2020; pp. 7689–7693. IEEE

[15] Park DS, Chan W, Zhang Y, Chiu C-C, Zoph B, Cubuk ED, Le QV. Specaugment: A simple data augmentation method for automatic speech recognition. arXiv preprint arXiv:1904.08779 2019

[16] Liu F. An open access database for evaluating the algorithms of electrocardiogram rhythm and morphology abnormality detection. J Med Imaging Health Inform. 2018;8(7):1368–73.

[17] Zheng J, Zhang J, Danioko S, Yao H, Guo H, Rakovski C. A 12-lead electrocardiogram database for arrhythmia research covering more than 10,000 patients. Sci Data. 2020;7(1):1–8. 32051412 10.1038/s41597-020-0386-x PMC7016169

[18] Wagner P, Strodthoff N, Bousseljot R-D, Kreiseler D, Lunze FI, Samek W, Schaeffter T. Ptb-xl, a large publicly available electrocardiography dataset. Sci Data. 2020;7(1):1–15. 32451379 10.1038/s41597-020-0495-6 PMC7248071

[19] He K, Zhang X, Ren S, Sun J. Deep residual learning for image recognition. In: Proceedings of the IEEE conference on computer vision and pattern recognition, 2016; pp. 770–778

[20] Tan M, Le QV. Efficientnet: Rethinking model scaling for convolutional neural networks. arXiv preprint arXiv:1905.11946 2019;

[21] Huang G, Liu Z, Van Der Maaten L, Weinberger KQ. Densely connected convolutional networks. In: Proceedings of the IEEE conference on computer vision and pattern recognition, 2017; pp. 4700–4708

[22] Hannun AY, Rajpurkar P, Haghpanahi M, Tison GH, Bourn C, Turakhia MP, Ng AY. Cardiologist-level arrhythmia detection and classification in ambulatory electrocardiograms using a deep neural network. Nat Med. 2019;25(1):65. 30617320 10.1038/s41591-018-0268-3 PMC6784839

[23] Madry A, Makelov A, Schmidt L, Tsipras D, Vladu A. Towards deep learning models resistant to adversarial attacks. arXiv preprint arXiv:1706.06083 2017;

